# Antibacterial and Antifungal Sesquiterpenoids from Aerial Parts of *Anvillea garcinii*

**DOI:** 10.3390/molecules25071730

**Published:** 2020-04-09

**Authors:** Shagufta Perveen, Jawaher Alqahtani, Raha Orfali, Hanan Y. Aati, Areej M. Al-Taweel, Taghreed A. Ibrahim, Afsar Khan, Hasan S. Yusufoglu, Maged S. Abdel-Kader, Orazio Taglialatela-Scafati

**Affiliations:** 1 Department of Pharmacognosy, College of Pharmacy, King Saud University. P. O. Box 22452, Riyadh 11495, Saudi Arabia; jalqahtani@ksu.edu.sa (J.A.); rorfali@ksu.edu.sa (R.O.); hati@ksu.edu.sa (H.Y.A.); amaltaweel@ksu.edu.sa (A.M.A.-T.); tshehata@ksu.edu.sa (T.A.I.); 2 Department of Pharmacy, School of Medicine and Surgery, University of Naples Federico II, Via D. Montesano 49, 80131 Naples, Italy; 3 Department of Chemistry, COMSATS University Islamabad, Abbottabad Campus, Abbottabad-22060, Pakistan; afsarhej@gmail.com; 4 Department of Pharmacognosy, College of Pharmacy, Prince Sattam Bin Abdulaziz University, P.O. Box 173, Al-Kharj 11942, Saudi Arabia; h.yusufoglu@psau.edu.sa (H.S.Y.); mpharm101@hotmail.com (M.S.A.-K.); 5 Department of Pharmacognosy, Faculty of Pharmacy, Alexandria University, Alexandria 21215, Egypt

**Keywords:** *Anvillea garcinii*, Saudi medicinal plants, sesquiterpene lactones, antifungal activity

## Abstract

Two new sesquiterpenoids belonging to the guaiane, 4α,9α,10α-trihydroxyguaia-11(13)en-12,6α-olide (**1**), and germacrane, 9β-hydroxyparthenolide-9-*O*-β-D-glucopyranoside (**2**), classes have been isolated from the leaves of the Saudi medicinal plant *Anvillea garcinii* along with seven known compounds (**3**–**9**). The structures of the new metabolites were elucidated by spectroscopic analysis, including one-dimensional (1D) and two-dimensional (2D) Nuclear Magnetic Resonance (NMR) and high-resolution electrospray ionization mass spectrometry (HR-ESIMS). The antimicrobial properties of **1**–**9** were screened against seven different pathogenic microbes, and compounds **1**–**3** showed a potent antifungal activity.

## 1. Introduction

Traditional medicines are a powerful weapon for mankind and they have been used to treat several health disorders since ancient times. The last few decades have witnessed a renaissance in the use of natural products, and traditional medicine in general, to prevent or cure several ailments. While they are considered one of the possible options in several countries, they are practically the main therapeutic option in many developing countries, including those of the Arabian Peninsula.

Flora of Saudi Arabia is evidently and understandably not as rich and diverse as that of countries of the Mediterranean basin; however, it has a vital role for various ecosystems, especially in maintaining the environmental balance and stability [1]. The dominating plant family is Asteraceae, one of the largest plant families on the planet, which is a family that includes more than one thousand genera and twenty thousand species [2]. Asteraceae plants are well known for their biological and pharmacological effects, largely ascribable to the presence of phytochemicals belonging to polyphenol, flavonoid, and terpenoid classes [3].

*Anvillea* is probably the smallest genus of the Asteraceae family, since it includes only four species, distributed in a large area spanning from North Africa to Iran, including several Middle Eastern countries, such as Egypt, Palestine, and Saudi Arabia [4]. In Saudi Arabia, *Anvillea* genus is represented by the following two species: *A. garcinii* and *A. radiata*. *A. garcinii* DC (Arabic name Nuqd) is one of the most important ethnomedicinal plants used in the Arabian Peninsula region, indicated for symptomatic relief of various illnesses such as cold, gastrointestinal disorders, and respiratory system problems [3]. Traditionally, the dried plant is crushed, mixed with honey or date and olive oil, and used to treat cold symptoms [4].

Flavonoids and sesquiterpene lactones have been the predominant class of secondary metabolites obtained by phytochemical studies on *A. garcinii* [5,6,7,8,9,10,11]. Our previous investigations on this plant disclosed the presence of sesquiterpene lactones of the guaianolide- and germacranolide-types, including the corresponding amino acid adducts, as well as some flavonoids glycosides [11,12,13]. The class of *Anvillea* sesquiterpene lactones is dominated by derivatives with the parthenolide skeleton, germacranolides endowed with significant biological activities in cancer and inflammation, as well as in metabolic disorders [14]. Previous examination of the aerial parts of *A. garcinii* have afforded several members of the parthenolide class, such as 9α- and 9β-hydroxyparthenolide, 9α- and 9β-hydroxy-1β,10α-epoxyparthenolide, parthenolid-9-one, and its *cis*-isomer [15]. In addition, guaianolide-type sesquiterpenoids, a class of phytochemicals with a broad range of activities, including cytotoxic, antiprotozoal, and anti-inflammatory potential [16], also constitute prominent *A. garcinii* metabolites. Leucodin and zaluzanin C and their derivatives [17], as well as garcinamine E and other guaianes [13], have been isolated from this species.

**Figure 1:**
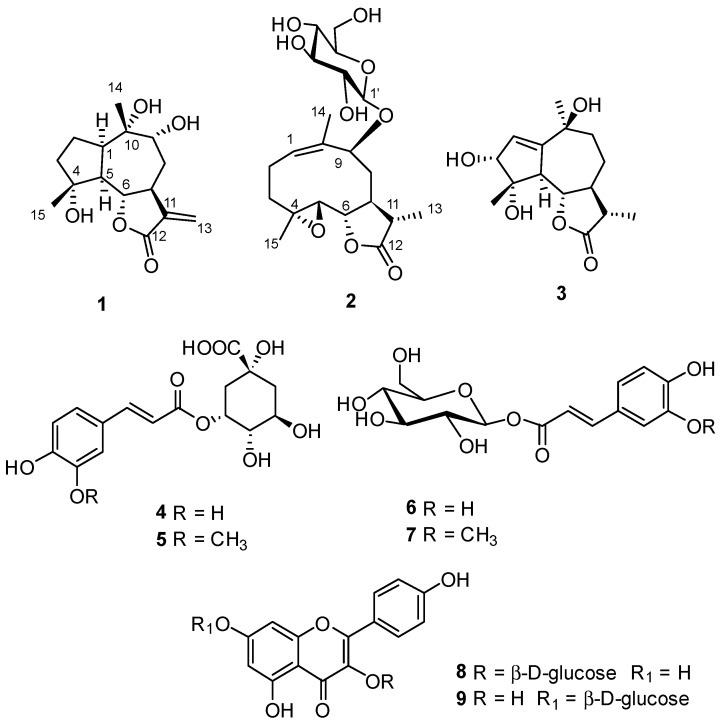
Chemical structures of metabolites isolated from *Anvillea garcinia*.

This richness of bioactive secondary metabolites prompted us to continue our phytochemical and biological investigation of *A. garcinii*, with the final aim of obtaining a detailed picture of the metabolome of this plant, providing solid scientific grounds for its use in traditional medicines and possibly, to develop a new phytotherapeutic drug from this natural source. In this manuscript we report the isolation of two new sesquiterpenoids belonging to the guaiane (4α,9α,10α-trihydroxyguaia-11(13)en-12,6α-olide, (**1**)) and germacranolide (9β-hydroxyparthenolide-9-*O*-β-D-glucopyranoside, (**2**)) classes, along with seven known compounds (**3**–**9**) (Figure 1) and the results of screening for antimicrobial activity on the isolated metabolites.

## 2. Results and Discussion

Previous investigations on *A. garcinii* aerial parts selected the *n*-butanol fraction of the methanol extract as the richest in polar sesquiterpenoids. Its chromatographic separation was achieved using a combination of Sephadex LH-20, silica gel, and RP-18 column chromatography, and yielded two new (**1**–**2**) and seven known compounds (**3**–**9**) (Figure 1). The structures of these metabolites were elucidated by spectroscopic analysis, mainly one-dimensional (1D) and two-dimensional (2D) NMR and electrospray ionization mass spectrometry (ESIMS). Compounds **3**–**9** were identified as 3α,4α,10β-trihydroxy-11β-guai-1-en-12,6α-olide (**3**) [18], chlorogenic acid (**4**) [19], 3-*O*-feruloylquinic acid (**5**) [20], 1-*O*-caffeoyl-β-D-glucopyranose (**6**) [21], 1-*O*-feruloyl-β-D-glucopyranose (**7**) [22], kaempferol-3-*O*-glucopyranoside (**8**) [23], and kaempferol-7-*O*-glucopyranoside (**9**) [24] by a comparison of their spectroscopic data with those reported in the literature. Selected spectra of these compounds are reported as Supplementary Materials. All these phenolic derivatives are reported from *A. garcinii* for the first time.

Compound **1** was isolated as a white solid with the molecular formula C_15_H_22_O_5_, determined by high-resolution electrospray ionization mass spectrometry (HR-ESIMS) (*m/z* 281.1400 [M–H]^−^; calculated for C_15_H_21_O_5_, 281.1394), indicating five unsaturation degrees. The ^1^H NMR spectrum of **1** showed the presence of a pair of sp^2^ methylene protons (δ_H_ 5.90 and 5.36, each bs); two oxygenated methine protons at δ_H_ 3.58 (1H, d, *J* = 2.5 Hz) and 4.17 (1H, dd, *J* = 9.5, 11.0 Hz); three relatively deshielded methines at δ_H_ 2.00 (1H, brd, *J* = 11.0 Hz), 2.87 (overlapped), and 2.84 (overlapped); three methylene protons, and two methyl singlets at δ_H_ 0.95 and 1.10 (see Table 1). The ^13^C NMR spectral data of **1**, which was analyzed with the help of the 2D NMR HSQC spectrum, disclosed the presence of one ester carbonyl at δ_C_ 170.8; one sp^2^ methylene at δ_C_ 118.7, and an additional unprotonated sp^2^ carbon at δ_C_ 139.8; two oxymethine carbons at δ_C_ 82.6 and 76.0 and two unprotonated oxygenated carbons at 79.6 and 76.6. The remaining carbon atoms were assigned as three sp^3^ methylenes at δ_C_ 24.6, 31.6, and 40.3, three sp^3^ methines at δ_C_ 40.5, 40.7, and 54.6, and two methyl carbon atoms at δ_C_ 21.7 and 22.0.

**Figure 2:**
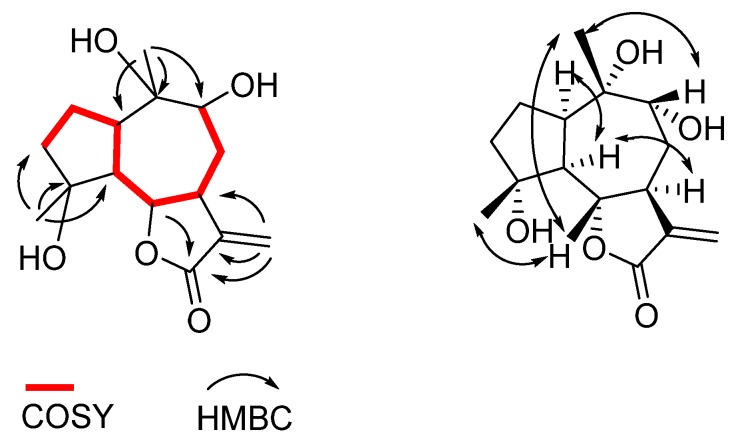
Some key two-dimensional (2D) NMR correlations of **1** (left, correlated spectroscopy (COSY) and heteronuclear multiple bond coherence (HMBC) and right, nuclear Overhauser enhancement spectroscopy (NOESY)).

The guaianolide-type skeleton of compound **1** was assembled on the basis of the 2D NMR COSY and heteronuclear multiple bond coherence (HMBC) spectra. The COSY spectrum disclosed the single extended spin system (highlighted in red in Figure 2), which was arranged on the bicyclic system on the basis of the HMBC correlations from methyl singlets H_3_-14 and H_3_-15. The HMBC correlations from the methylene H-13 to C-12, the nuclear Overhauser enhancement spectroscopy (NOESY) correlations H-6/H_3_-15, H-6/H_3_-14, and H-9/H_3_-14, and the remaining NOESY correlation of **1,** shown in Figure 2, indicated the relative configuration of compound **1**. Thus, using all the above-mentioned data, compound **1** was elucidated as 4α,9α,10α-trihydroxyguaia-11(13)-en-12,6α-olide.

Interestingly, the presence of **1** as a component of the sesquiterpenoid pool of *A. garcinii* was anticipated by us at the time of isolation of garcinamine E [13]. Indeed, garcinamine E, co-occurring in the *n*-butanol fraction of the leaves of *A. garcinii*, is the L-proline adduct at C-13 of **1**.

Compound **2** was obtained as an optically active ([α]^25^ D = −55, *c* = 0.10, CH_3_OH) yellow gummy solid with molecular formula C_21_H_32_O_9_, as established by HR-ESIMS. The ^13^C NMR spectrum, which was interpreted taking into account data from the 2D NMR HSQC and HMBC experiments, indicated the presence of one ester carbonyl (δ_C_ 178.7); two olefinic carbons (δ_C_ 129.4 and 133.9); one anomeric carbon (δ_C_ 98.7); seven additional oxygenated methines (δ_C_ 83.1, 81.4, 76.7, 76.5, 73.5, 70.3 and 65.9); one oxygenated unprotonated carbon (δ_C_ 61.8); one oxygenated methylene (δ_C_ 61.4); and eight additional sp^3^ carbons, including two methines, three methylenes, and three methyls (Table 1). Accordingly, the ^1^H NMR spectrum of **2** (Table 1) showed a *sp*^2^ methine signal at δ_H_ 5.56, a series of oxymethine and oxymethylene protons between δ_H_ 4.37 and 3.17, and three methyl signals, namely a deshielded singlet at δ_H_ 1.76, a singlet at δ_H_ 1.36, and a doublet at δ_H_ 1.27. These data were indicative of the sesquiterpene lactone glycoside nature for compound **2**, whose structure was assigned on the basis of a detailed inspection of the 2D NMR correlations and comparison with data of known compounds [12].

In particular, the COSY spectrum of **2** revealed the presence of three spin systems, namely (i) from the sp^2^ methine H-1 to H_2_-3, (ii) from H-5 to H-9 including the H-11/H_3_-13 branching, and (iii) the sugar spin system (H-1′ to H_2_-6′). The sugar unit was assigned as a β-D-glucopyranoside on the basis of the coupling constant H-1′/H-2′ (*J* = 7.5 Hz) and acid hydrolysis of **2**, which afforded the free sugar unit, identified as D-glucose through co-TLC and optical rotation sign.

The HMBC correlations of **2** were instrumental to join the above deduced fragments. In particular, ^3^*J* cross-peaks of H_3_-14 with C-1, C-9, and the unprotonated sp^2^ C-10 and of H_3_-15 with C-3, C-4, and C-5 indicated the presence of the ten-membered ring. Moreover, cross-peaks of both H-6 and H_3_-13 with the ester carbonyl carbon C-12 indicated the presence of the lactone ring. The linkage of the D-glucopyranose moiety at position 9 was indicated by the ^3^*J* HMBC correlation of H-1′ (δ_H_ 4.10) with C-9 (δ_C_ 83.1). The presence of an epoxyde ring at C-4/C5, which accounted for the remaining unsaturation degree, was in perfect agreement with the NMR resonances of the involved carbons (C-4 = 61.8 ppm and C-5 = 65.9 ppm). The NOESY correlations were, then, used to deduce the relative configuration of compound **2**. In particular, NOESY cross-peak of H-1 with H_3_-14 indicated the *Z* configuration of the endocyclic double bond. The relative configuration of the five consecutive stereogenic centers (C-4 to C-11) was deduced on the basis of the following NOESY correlations: H_3_-15/H-6, H-5/H-7, H_2_-3/H-5, and H-7/H_3_-13. Finally, the NOESY cross-peak of H-7 with H-9 pointed to the α-orientation of both protons. Thus, compound **2** was identified as the new 1Z-9β-hydroxyparthenolide-9-*O*-β-D-glucopyranoside. The Δ^1,10^-*E* isomer of **2** has been recently isolated from *Asteriscus graveolens* [25] and, accordingly, its reported ^13^C NMR resonances for C-1 and C-10 showed significant differences as compared with those of **2**. Both compounds **1** and **2** are close analogues of sesquiterpenoids previously isolated from the same species and we have assumed that they share the absolute configuration of their co-occurring analogues.

The isolated metabolites (**1–9**) were evaluated for their antimicrobial activity against pathogenic bacteria and fungi (Table 2 and Table 3). Compounds **1**–**3** showed antifungal activities against human pathogenic fungi, with a growth inhibitory activity around 80% at 50 μg mL^−1^ against *Candida albicans* and *C. parapsilosis,* respectively. The respective minimum inhibitory concentrations (MIC) of **1**–**3** were 0.21, 0.26, and 0.38 µg mL^−1^ against *C. albicans* and 0.25, 0.31, and 0.34 against *C. parapsilosis*. This finding is in agreement with our previously reported results on antifungal activity of guaianolide sesquiterpenoids [26]. In addition, **1–3** also showed activity against Gram-positive and Gram-negative pathogenic bacteria with MIC ranging from 2.3 to 6.3 μg mL^−1^ (Table 3). Chlorogenic acid (**4**), a non-sesquiterpenoid, showed a significant inhibition against pathogenic fungi (Table 2) and strong antibacterial activity against the Gram-negative bacteria *E. xiangfangensis* and *E. fergusonii*. Ester derivatives **6** and **7** showed neither antifungal nor antibacterial activity at 25 μg mL^−1^.

## 3. Materials and Methods

### 3.1. General

Optical rotations were measured in analytical grade methanol using a JASCO P-2000 polarimeter (JASCO, 2967-5, Tokyo, Japan). The 1D and 2D NMR data were acquired using a Bruker AVANCE spectrometer (Bruker, Billerica, MA, USA) (500 MHz for ^1^H and 125 MHz for ^13^C). Chemical shifts (δ) in ppm, relative to tetramethylsilane, were calculated basing on the residual solvent signal, and *J* scalar coupling constants are reported in Hz (Hertz). The ESI-MS analyses were measured on an Triple Quadrupole 6410 QQQ LC/MS mass spectrometer (Agilent, Santa Clara, CA, USA) with ESI ion source (gas temperature was 350 °C, nebulizer pressure was 60 psi, and gas flow rate was 12 L/min), operating in the negative and positive scan modes of ionization through direct infusion method using CH_3_OH\H_2_O (4:6 *v/v*) at a flow rate of 0.5 mL/min. Column chromatography procedures were performed using silica gel 70–230 mesh, RP-18, Sephadex LH-20 (each from; E. Merck, Darmstadt, Germany). TLC analysis was performed using precoated silica gel 60 F_254_ and RP-18 (Merck, Darmstadt, Germany) plates, and spots were visualized via exposure under UV light (254/365 nm) and by spraying with different spray reagent. Analytical grade solvents and reagents were purchased from Sigma-Aldrich (St. Louis, MO, USA). Deuterated methanol (CD_3_OD-*d*) and dimethylsulfoxide (DMSO-*d*_6_) were obtained from Cambridge Isotope Laboratories (Tewksbury, MA, USA).

### 3.2. Plant Material

The aerial parts of *A. garcinii* were collected in March 2018 in the area 17 km South West of Al-Kharj city and identified by taxonomist, Dr. M. Atiqur Rahman, College of Pharmacy, Medicinal, Aromatic and Poisonous Plants Research Center, King Saud University. A voucher specimen (PSAU-CPH-6-2018) is kept in the herbarium of College of Pharmacy, Prince Sattam Bin Abdulaziz University.

### 3.3. Extraction and Isolation

The shade dried powdered aerial parts of *A. garcinii* (0.5 kg) were extracted with methanol at room temperature (3 × 2.5 L). Total methanol extract was concentrated under reduced pressure using a rotary evaporator (Büchi Rotavapor RII, Flawil, Switzerland). The crude extract (50 g) was suspended in H_2_O (0.5 L) and extracted successively with chloroform and *n*-butanol, and then the residual water fraction was lyophilized. The *n*-butanol soluble fraction (30 g) was subjected to a silica gel open column and eluted with a gradient of CH_2_Cl_2_:CH_3_OH (9.5:0.5→1.0:9.0), to afford nine major fractions 1–9 based on their TLC image. Fraction 3 (0.8 g) was further subjected to RP C-18 column chromatography, eluted under medium pressure with a gradient of water/methanol (4.0:6.0→9.0:1.0), to obtain two subfractions which was further loaded on a RP C-18 column and eluted with a gradient mixture of water/methanol (6.0:4.0→1.0:9.0), which yielded compounds **1** (10 mg) and **3** (8 mg). Fraction 5 (0.3 g) was applied to a RP C-18 column using water/methanol (8.0:2.0→1.0:1.0) to yield compound **2** (15 mg). Fraction 6 (0.6 g) was rechromatographed on a Sephadex LH-20 column with water/methanol (1:1–100:0) to afford two compounds **4** (10 mg) and **5** (12 mg). Fraction 7 (0.5 g) was separated on a Sephadex LH-20 column with a gradient mixture of water/methanol (9.0:1.0→7.0:3.0), and finally was divided on a silica gel column with CHCl_3_/MeOH (8.5:1.5) to afford compounds **6** (6.0 mg) and **7** (11 mg). Subfraction 8 (0.2 g) was further purified by HPLC (flow rate 1 mL/min, wavelength 254 nm, and CH_3_OH–0.01%HCOOH/H_2_O, 4:6) to afford **8** (15 mg, Rt 25.5 min). Subfraction 9 (0.1 g) was purified by HPLC (flow rate 1.0 mL/min, wavelength 254 nm, CH_3_OH–0.01%HCOOH/H_2_O, 1:1) to afford compound **9** (12 mg, Rt 27.5 min).

4α,9α,10α-Trihydroxyguaia-11(13)en-12,6α-olide (**1**): Yellow gummy solid, [α]_D_^25^ + 72 (c 0.10, MeOH); UV (MeOH), λ_max_ 223 nm (ε 4000); ^1^H NMR (500 MHz, in CD_3_OD) and ^13^C NMR (125 MHz, in CD_3_OD) see Table 1; negative ions ESIMS, *m/z* 281.1400 [M–H]^−^, calculated for C_15_H_21_O_5_, 281.1394.

1*Z*-9β-Hydroxyparthenolide-9-*O*-β-D-glucopyranoside (**2**): Yellow gummy solid, [α]_D_^25^ + 55 (c 0.10, MeOH); UV (MeOH), λ_max_ 211 nm (ε 3200); ^1^H NMR (500 MHz, in CD_3_OD) and ^13^C NMR (125 MHz, in CD_3_OD) see Table 1; positive ions ESIMS, *m/z* 451.1948 [M+ Na]^+^, calculated for C_21_H_32_NaO_9_, 451.1944.

### 3.4. Acid Hydrolysis of 2

Compound **2** (3.0 mg) was dissolved in 0.6 mL of a solution of 1N HCl–methanol (1:1). The mixture was heated at 65 °C for 60 min and concentrated in vacuo, water was added and the whole was extracted with ethyl acetate. The aqueous portion was filtered, the filtrate was evaporated, and D-glucose (0.9 mg, 71%) was identified from the sign of its optical rotation ([α]^25^_D_ + 52.0) and co-TLC (*n*-butanol/water/acetic acid, 8:2:10, *Rf* 0.17) with an authentic sample of D-glucose (Merck) using anisaldehyde as spray reagent for visualization.

### 3.5. Antibacterial Bioassay

The antibacterial activity was determined according the reported method [27]. Muller Hinton agar plate contained microorganisms after suspension in a nutrient broth for 24 h wells were created in the plate and loaded with 10 µL of the sample solution obtained using DMSO as solvent. Amikacin was used as standard antibiotic for five different pathogenic bacteria two were Gram-positive, i.e., *Staphylococcus aureus* (CP011526.1) and *Bacillus licheniformis* (KX785171.1) and three were Gram-negative, i.e., *Enterobacter xiangfangensis* (CP017183.1), *Escherichia fergusonii* (CU928158.2), and *Pseudomonas aeruginosa* (NR-117678.1).

The clear area which was free of microbial growth was measured three times to detect the diameter of the zone of inhibition and the mean were recorded. The minimal inhibitory concentration (MIC, μg mL^−1^) of the tested isolated compounds that inhibited the visible bacterial growth was calculated using varying concentrations of the tested compounds following the broth microdilution method [27,28].

### 3.6. Antifungal Assay

Well diffusion and broth microdilution techniques were used in this study to detect the antifungal activity of the isolated compounds. According to Gong and Guo [29], in SDA plate the sample solutions (100 µL), approximately 3 × 10^6^ colony-forming units (CFU) mL^−1^ was smeared of two pathogenic fungi, *Candida albicans* and *Candida parapsilosis*. Wells were created in SDA plates and loaded with the 10 µg of the tested compounds dissolved in DMSO and incubated at 37 °C for 1 day. Itraconazole was used as a standard antifungal and the diameters (in mm) of zone of inhibition were measured. The rates of growth inhibition were obtained according to the following formula taking into consideration ± SD as means:

% Growth inhibition rate = (*d*_c_ − *d*_s_) / (*d*_c_ − *d*_0_) × 100

where *d*_c_ is the diameter of the untreated control fungus, *d*_s_ is the diameter of the sample-treated fungus, and *d*_0_ is the diameter of the fungus cut.

The minimal inhibitory concentration (MIC) of the isolated compounds **1**–**9** against *Candida albicans* and *C. parapsilosis* was determined by using varying concentrations of the tested compounds following the broth microdilution method following the instructions of the Clinical and Laboratory Standards Institute. Serial dilution of the isolated compounds was prepared into two-fold using sterile Roswell Park Memorial Institute (RPMI) 1640 medium with MOPS (0.165 mol L-1) and presence of glucose (2%). The 96-well microplates were performed and incubated at 37 °C for 24 h.

### 3.7. Statistical Analysis

Data analysis was expressed as mean ± standard deviation (SD) of three replicates. Where applicable, the data were subjected to one-way analysis of variance (ANOVA). According to a Microsoft Excel 2010 statistical package analyses, the significant differences were considered statistically significant P values < 0.05.

## 4. Conclusions

This investigation on the Saudi Arabia plant *A. garcinii* yielded two additional members of the sesquiterpene lactone class, a new guaianolide, anticipated as precursor of garcinamine E, and a new parthenolide glycoside. Both of these compounds have been evidenced to have a significant antifungal activity, complementing that which had already been revealed for previously isolated congeners of the same family. Therefore, this study clearly evidences the potential of even a single plant to provide a countless list of bioactive phytochemicals and, more in general, of the Saudi Arabian flora to enrich the global phytochemical effort. Although less diverse than others, Saudi Arabian flora is well worth of being studied in detail, also to provide scientific basis to the traditional use of medicinal plants in this area.

## Figures and Tables

**Table 1:** ^1^H (500 MHz) and ^13^C (125 MHz) NMR data for compounds **1** and **2** in CD_3_OD.

1			2	
Pos.	δ_H_ (mult., *J* in Hz)	δ_C_	δ_H_ (mult., *J* in Hz)	δ_C_
1	2.80 (m)	40.5	5.56 (dd, 9.5, 11.5)	129.4
2α	1.64 (dddd, 1.5, 3.5, 7.5, 12.5)	24.6	2.61 (m)	23.5
2β	1.49 (tdd, 2.0, 6.5, 12.5)		2.22 (m)	
3	1.53 (m)	40.4	2.13 (m)	35.9
			1.22 (m)	
4	-	79.6	-	61.8
5	2.00 (t, 11.0)	54.6	2.79 (d, 9.0)	65.9
6	4.17 (dd, 9.5, 11.0)	82.6	4.04 (t, 9.0)	81.4
7	2.87 (ddd, 9.5, 3.5, 1.5)	40.7	2.11 (m)	48.2
8 α	1.99 (ddd, 2.5, 3.5, 9.2)	31.6	2.09 (dd, 2.1, 9.2)	35.0
8β	1.53 (ddd, 1.0, 1.5, 9.2)	-	1.98 (bdd, 9.2, 7.5)	-
9	3.58 (dd, 2.5, 1.0)	76.0	4.37 (dd, 7.5, 2.1)	83.1
10	-	76.6	-	133.9
11	-	139.8	2.49 (dt, 2.5, 7.0)	41.7
12	-	170.8	-	178.7
13a	5.90 (brs)	118.7	1.27 (d, 7.0)	12.0
13b	5.36 (brs)	-		
14	0.95 (s)	21.8	1.76 (s)	10.0
15	1.10 (s)	22.0	1.36 (s)	16.3
1′			4.11 (d, 7.5)	98.7
2′			3.23 (t, 7.5)	73.5
3′			3.17 (m)	76.5
4′			3.29 (m)	70.3
5′			3.31 (m)	76.7
6′a			3.67 (m)	61.4
6′b			3.86 (m)	

**Table 2:** Antifungal activity of compounds **1**–**9**.

Compound	Growth Inhibition (%, mean ± SD) *		MIC (µg mL^−1^)	
	*C. albicans*	*C. parapsilosis*	*C. albicans*	*C. parapsilosis*
**1**	83.4 ± 3.3	81.3 ± 2.6	0.21 ± 0.04	0.25 ± 0.05
**2**	79.8 ± 5.3	76.5 ± 4.5	0.26 ± 0.07	0.31 ± 0.02
**3**	85.0 ± 3.4	80.0 ± 2.7	0.38 ± 0.03	0.34 ± 0.06
**4**	61.2 ± 3.3	69.5 ± 2.4	0.89 ± 0.02	0.61 ± 0.08
**5**	23.6 ± 5.2	18.9 ± 3.7	0.68 ± 0.01	0.79 ± 0.03
**6**	19.5 ± 2.9	21.7 ± 3.4	0.73 ± 0.08	0.86 ± 0.07
**7**	15.8 ± 3.2	10.9 ± 4.7	0.97 ± 0.12	0.79 ± 0.06
**8**	42.7 ± 4.4	51.8 ± 2.5	0.74 ± 0.05	0.62 ± 0.03
**9**	45.3 ± 3.7	49.9 ± 4.8	0.68 ± 0.08	0.74 ± 0.02
Itraconazole	54.7 ± 2.6	51.5 ± 4.1	0.29 ± 0.06	0.33 ± 0.04

* Results expressed as mean ± standard deviation (SD).

**Table 3:** Antibacterial activity of compounds **1–9**.

Compound	MIC (µg mL^−1^)				
	*Staphilococcus aureus*	*Bacillus licheniformis*	*Escherichia xiangfangensis*	*Escherichia fergusonii*	*Pseudomonas aeruginosa*
**1**	2.3	2.3	>25	5.7	>25
**2**	3.4	3.1	>25	6.3	>25
**3**	5.2	4.4	3.8	>25	>25
**4**	>25	>25	5.2	4.6	>25
**5**	>25	>25	>25	>25	>25
**6**	>25	>25	>25	>25	>25
**7**	>25	>25	>25	>25	>25
**8**	9.4	>25	>25	6.8	>25
**9**	>25	7.5	>25	8.4	>25
Amikacin	0.523	0.523	0.523	0.523	0.523

* Results expressed as mean ± standard deviation (SD).

## Supplementary Materials

The following are available online at https://www.mdpi.com/1420-3049/25/7/1730/s1. Figures S1 and S2: ^1^H and ^13^C NMR spectra of compound **1**. Figures S3 and S4: DEPT-90 and DEPT-135 of compound **1**. Figures S5 and S6: 2D NMR COSY and HSQC spectra of compound **1**. Figure S7: negative ESIMS spectrum of compound **1**. Figures S8 and S9: ^1^H and ^13^C NMR spectra of compound **2**. Figures S10 and S11: DEPT-135 and DEPT-90 of compound **2**. Figures S12 and S13: 2D NMR COSY and HSQC spectra of compound **2**. Figure S14: positive ESIMS spectrum of compound **2**. Figures S15 and S16: ^1^H and ^13^C NMR spectra of compound **3**. Figures S17 and S18: ^1^H and ^13^C NMR spectra of compound **4**. Figure S19: DEPT-90 of compound **4**. Figures S20 and S21: ^1^H and ^13^C NMR spectra of compound **5**. Figures S22 and S23: ^1^H and 2D HSQC spectra of compound **6**.
