# Supplementary material for: Antibacterial and Antifungal Sesquiterpenoids from Aerial Parts of Anvillea garcinii
Source: Molecules. 2020 Apr 9;25(7):1730. doi: 10.3390/molecules25071730 (PMC7180898; doi:10.3390/molecules25071730)
Supplement: Supplementary file 1 [file molecules-25-01730-s001.pdf]

Antibacterial and antifungal sesquiterpenoids from aerial parts of *Anvillea garcinii*

Shagufta Perveen[1,*], Jawaher Alqahtani[1,2], Raha Orfali[1], Hanan Y. Aati[1], Areej M. Al-Taweel[1], Taghreed A. Ibrahim[1], Afsar Khan[3], Hasan S. Yusufoglu[4], Maged S. Abdel-Kader[4,5] and Orazio Taglialatela-Scafati[2],*

Supplementary data

Figure S1. ^{1}H NMR Spectrum of Compound **1** in CD_3OD.
Figure S2. ^{13}C NMR Spectrum of Compound **1** in CD_3OD.
Figure S3. DEPT-90 NMR Spectrum of Compound **1** in CD_3OD.
Figure S4. DEPT-135 NMR Spectrum of Compound **1** in CD_3OD.
Figure S5. 2D COSY NMR Spectrum of Compound **1** in CD_3OD.
Figure S6. HSQC Spectrum of Compound **1** in CD_3OD.
Figure S7. Negative ESI Mass Spectrum of Compound **1**.
Figure S8. ^{1}H NMR Spectrum of Compound **2** in CD_3OD.
Figure S9. ^{13}C NMR Spectrum of Compound **2** in CD_3OD.
Figure S10. DEPT-135 NMR Spectrum of Compound **2** in CD_3OD.
Figure S11. DEPT-90 NMR Spectrum of Compound **2** in CD_3OD.
Figure S12. 2D COSY NMR spectrum of Compound **2** in CD_3OD.
Figure S13. HSQC Spectrum of Compound **2** in CD_3OD.
Figure S14. Positive ESI Mass Spectra of Compound **2**.
Figure S15. ^{1}H NMR spectrum of compound **3**.
Figure S16. ^{13}C NMR spectrum of compound **3**.
Figure S17. ^{1}H NMR spectrum of compound **4**.
Figure S18. ^{13}C NMR spectrum of compound **4**.
Figure S19. DEPT NMR spectrum of compound **4**.
Figure S20. ^{1}H NMR spectrum of compound **5**.
Figure S21. ^{13}C NMR spectrum of compound **5**.
Figure S22. ^{1}H NMR spectrum of compound **6**.
Figure S23. 2D HSQC NMR spectrum of compound **6**.

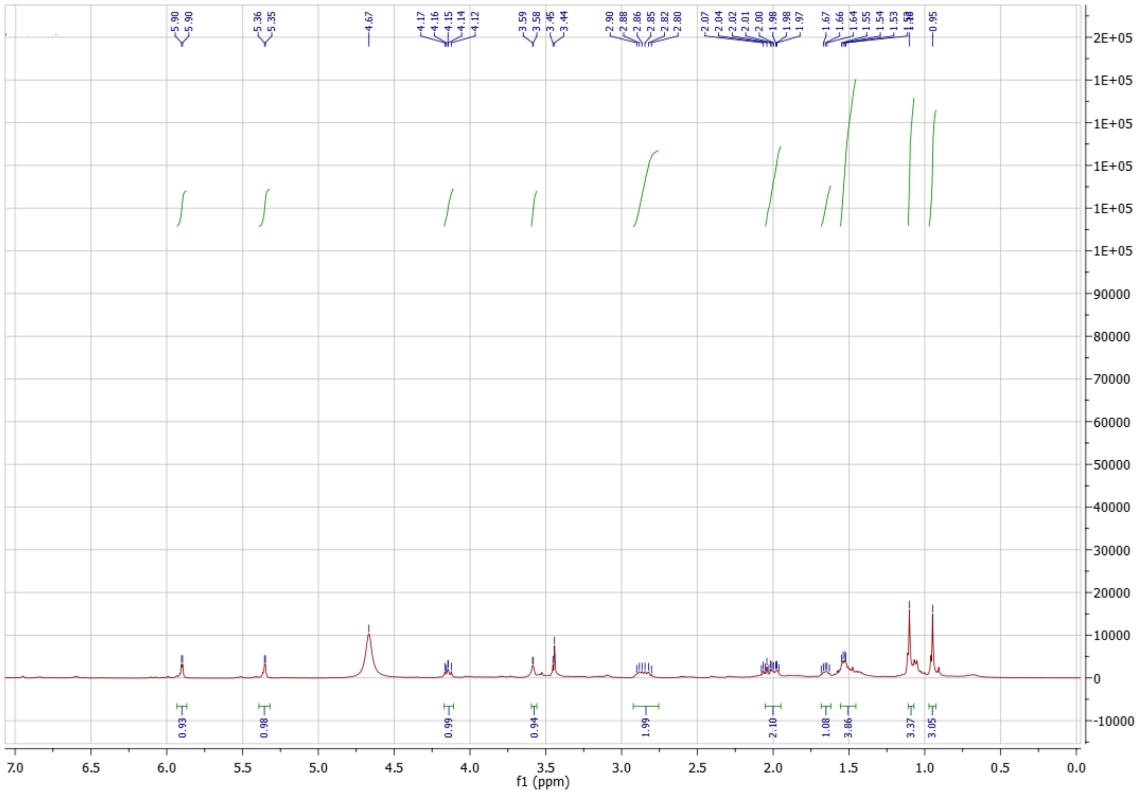

Figure S1. ^{1}H NMR spectrum of Compound **1** in CD_3OD.

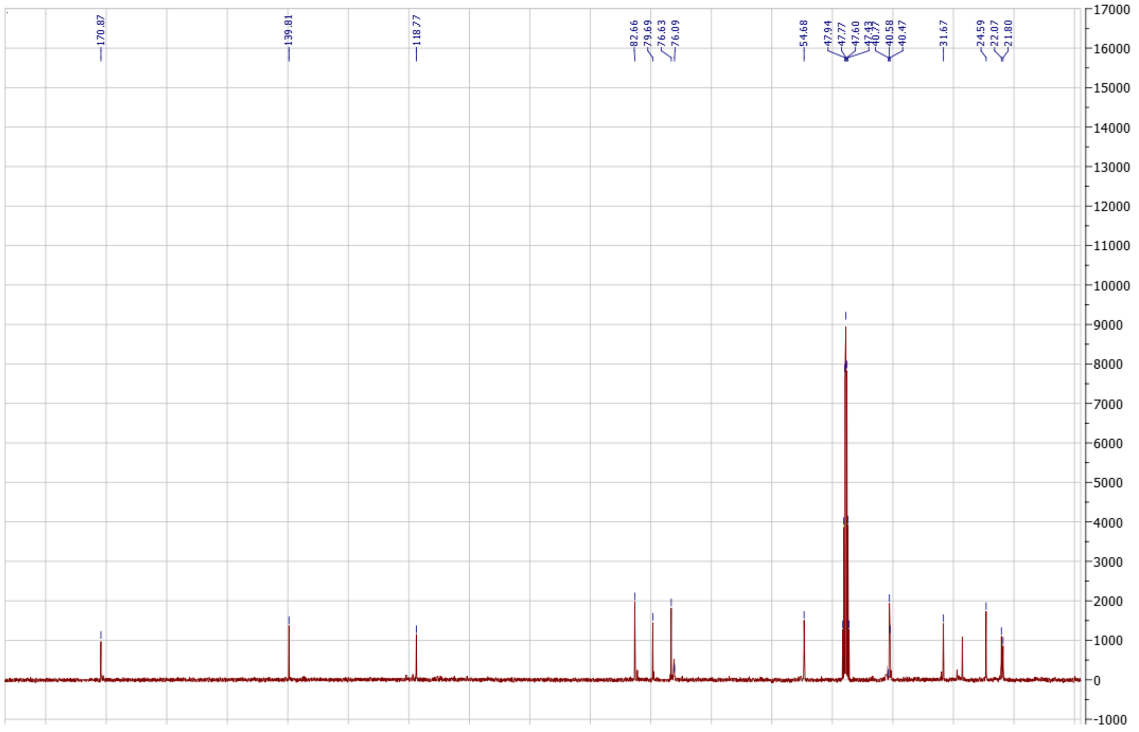

Figure S2. ^{13}C NMR spectrum of Compound **1** in CD_3OD.

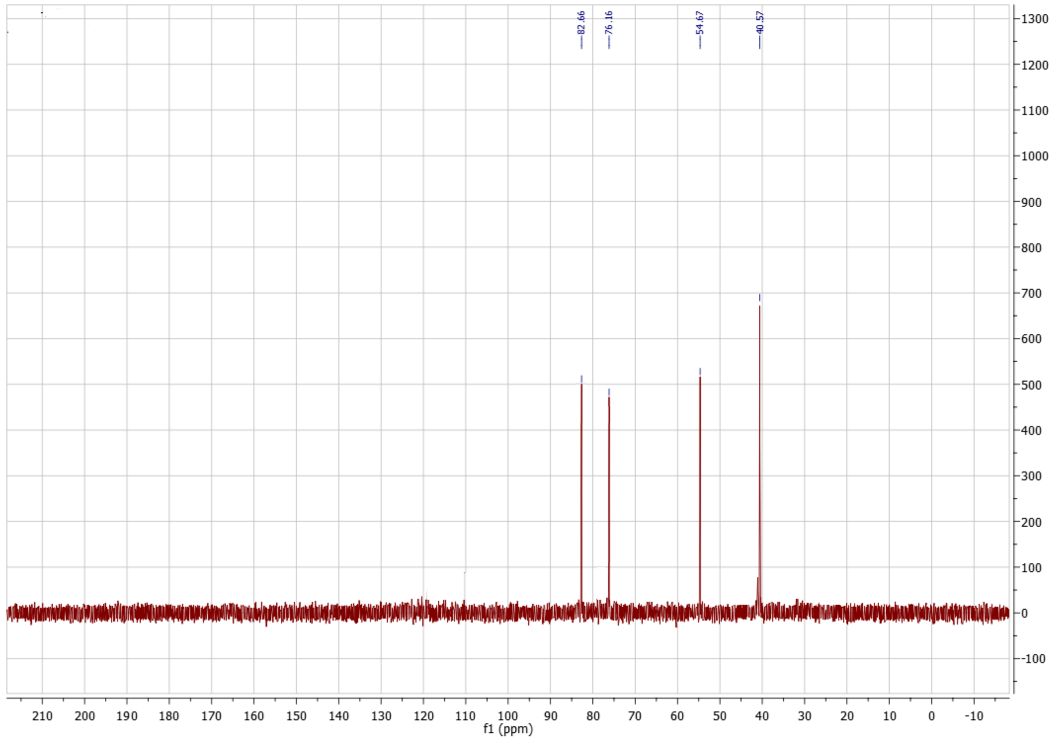

Figure S3. DEPT-90 NMR spectrum of Compound **1** in CD_3OD.

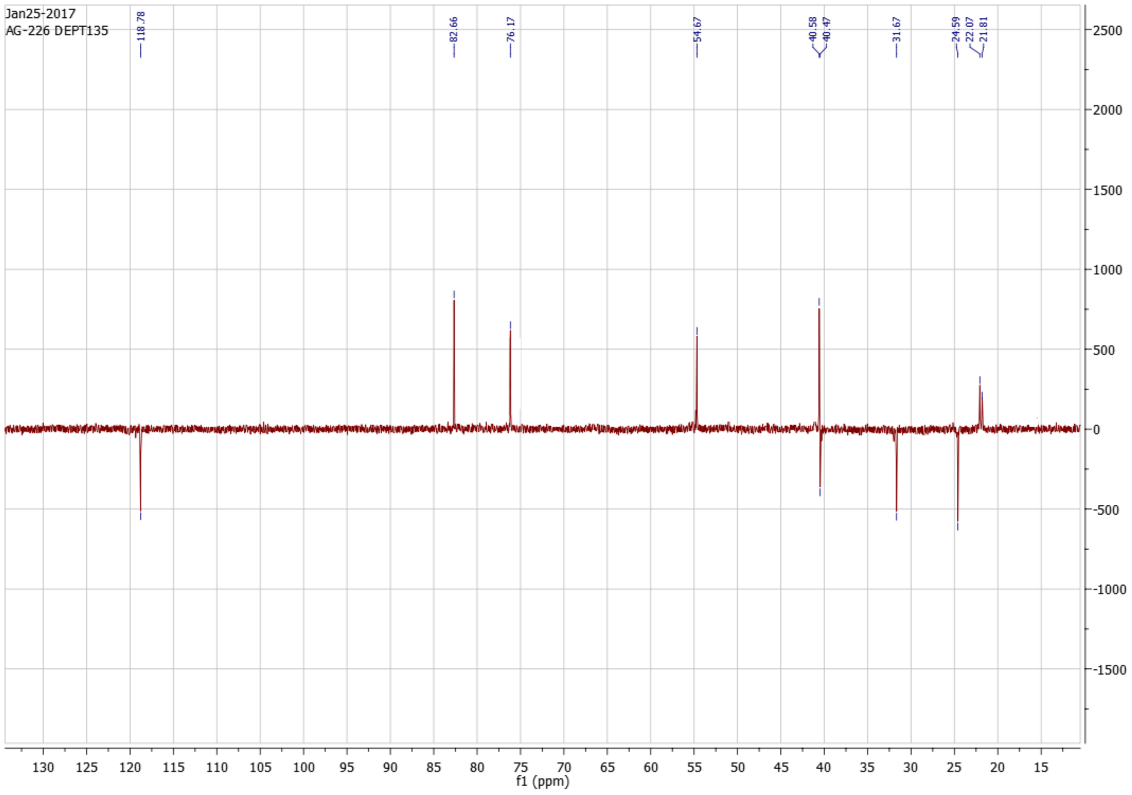

Figure S4. DEPT-135 NMR Spectra of Compound **1** in CD_3OD.

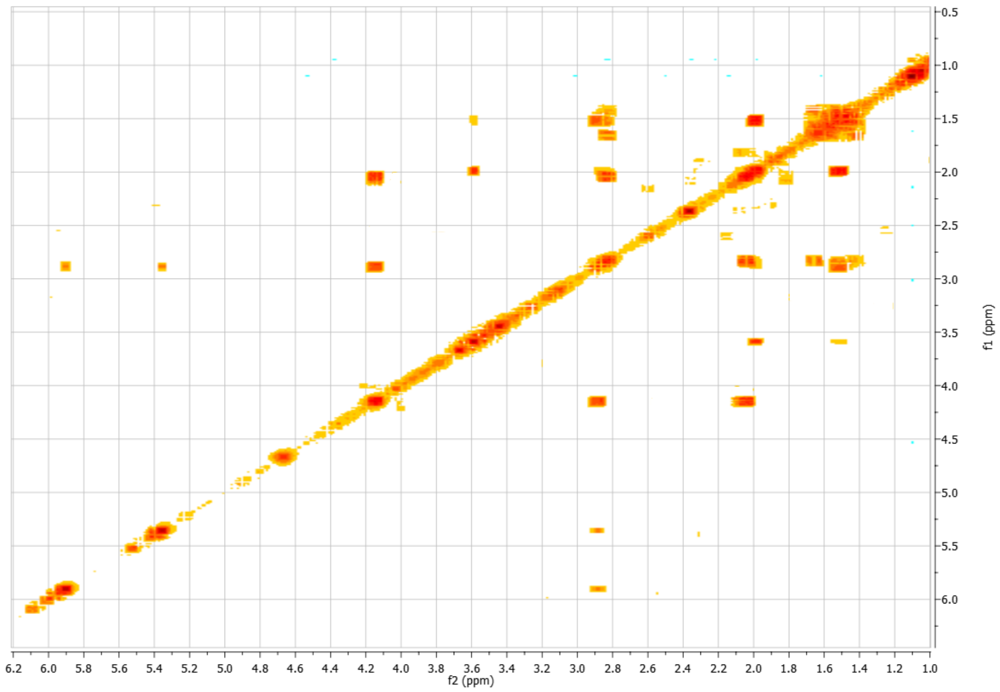

Figure S5. 2D COSY NMR Spectrum of Compound **1** in CD_3OD.

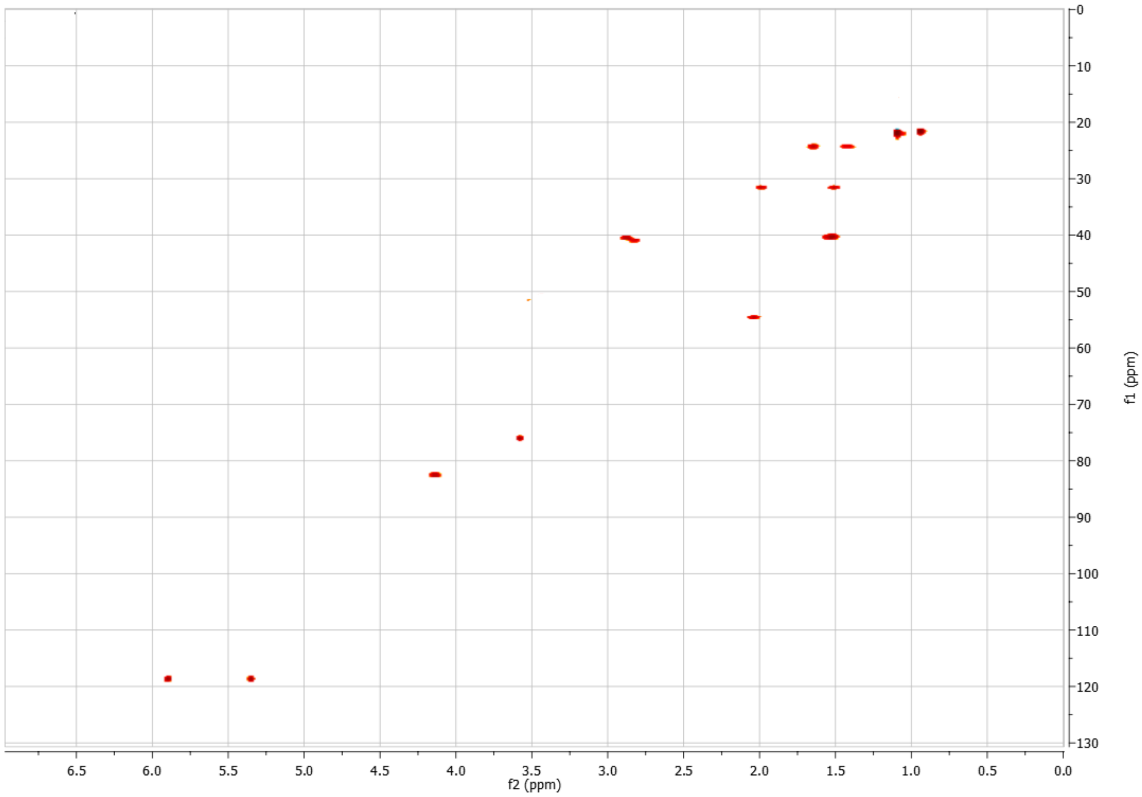

Figure S6. 2D NMR HSQC spectrum of Compound **1** in CD_3OD.

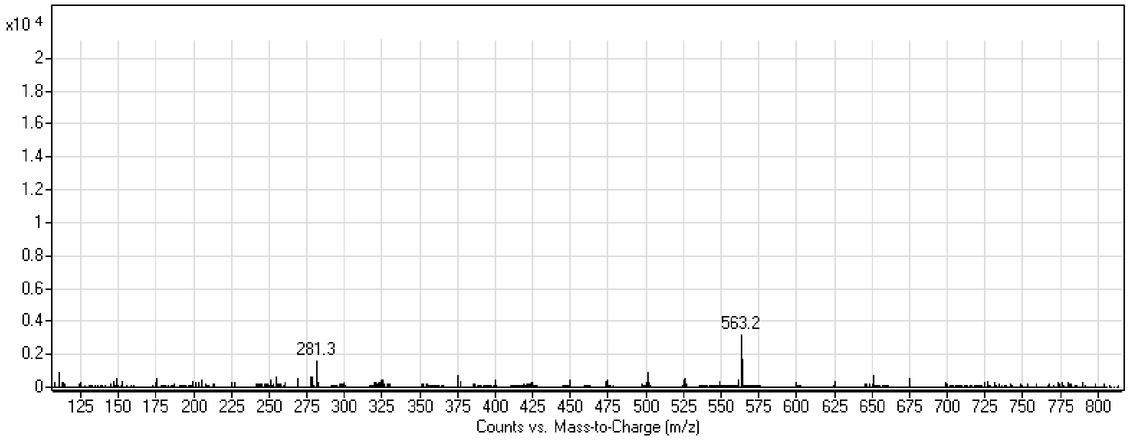

Figure S7. Negative-ions ESI Mass spectrum of Compound **1**.

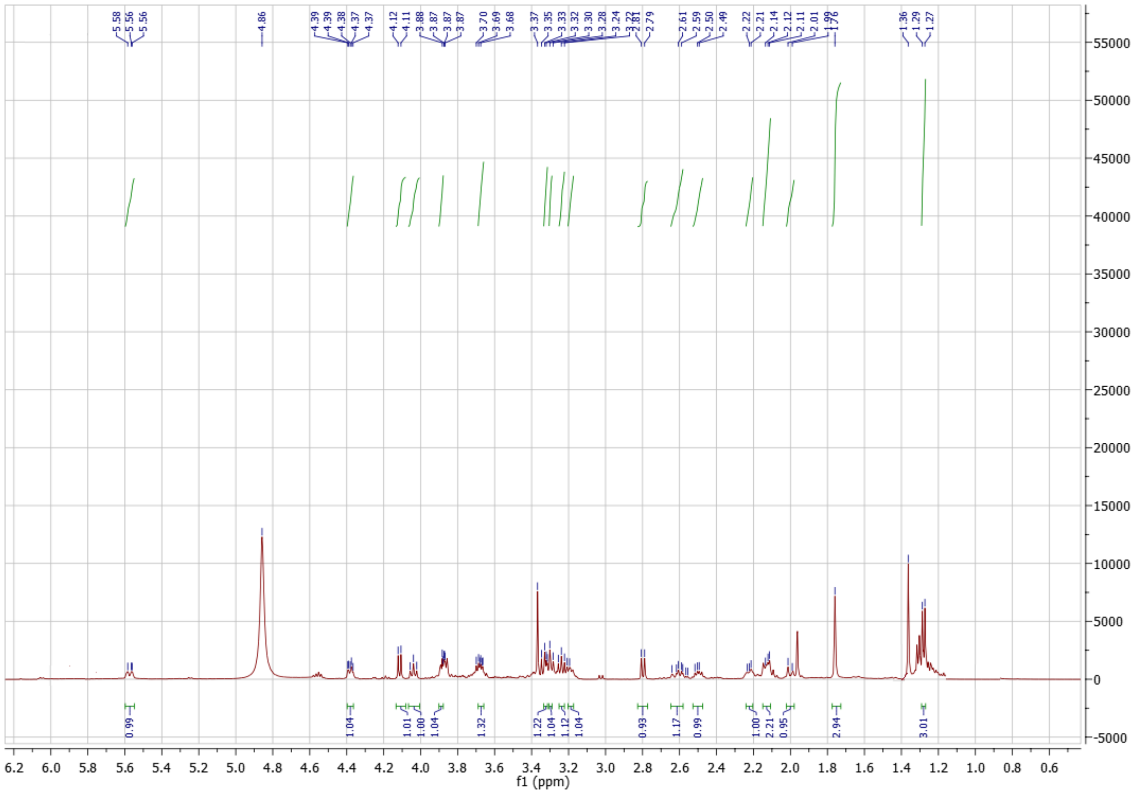

Figure S8. ^{1}H NMR spectrum of Compound **2** in CD_3OD.

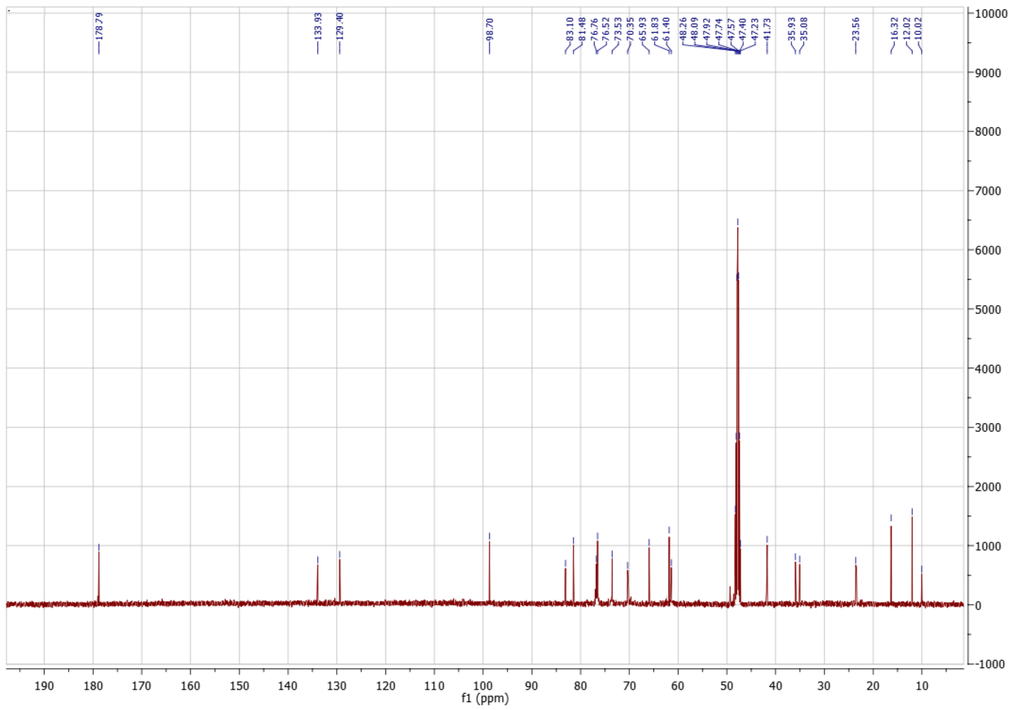

Figure S9. ^{13}C NMR spectrum of Compound **2** in CD_3OD.

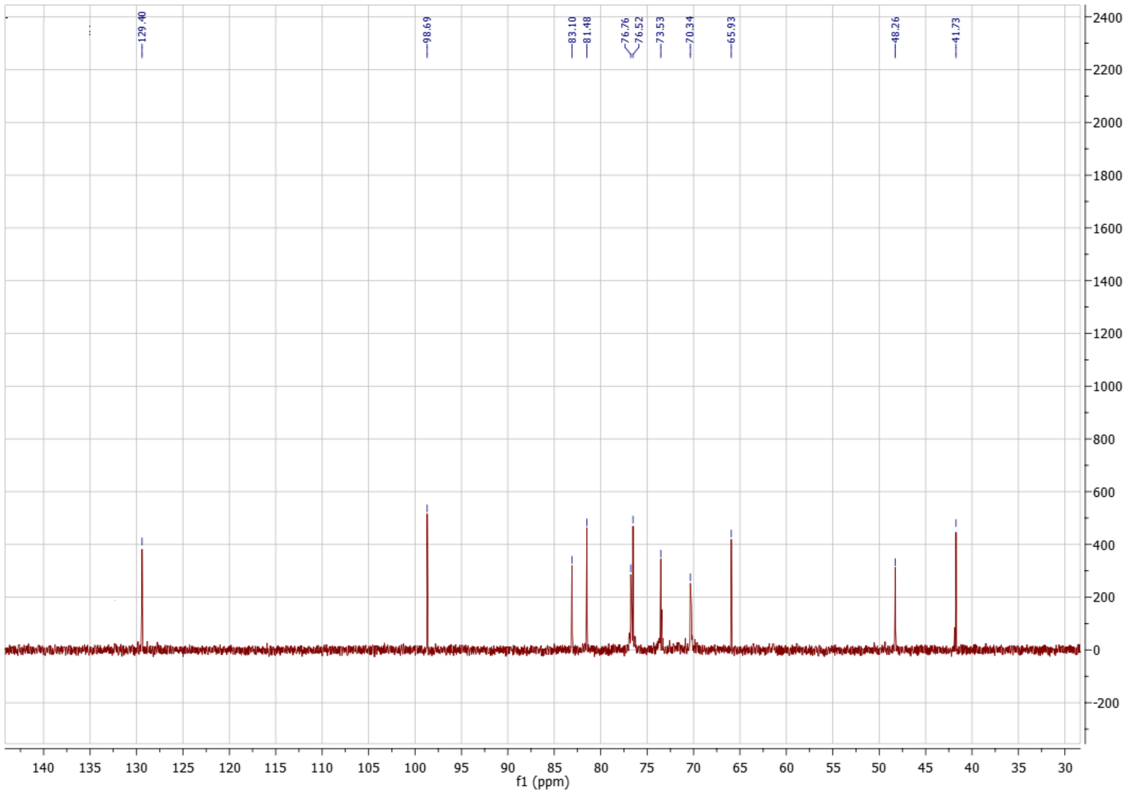

Figure S10. DEPT-90 NMR spectrum of Compound **2** in CD_3OD.

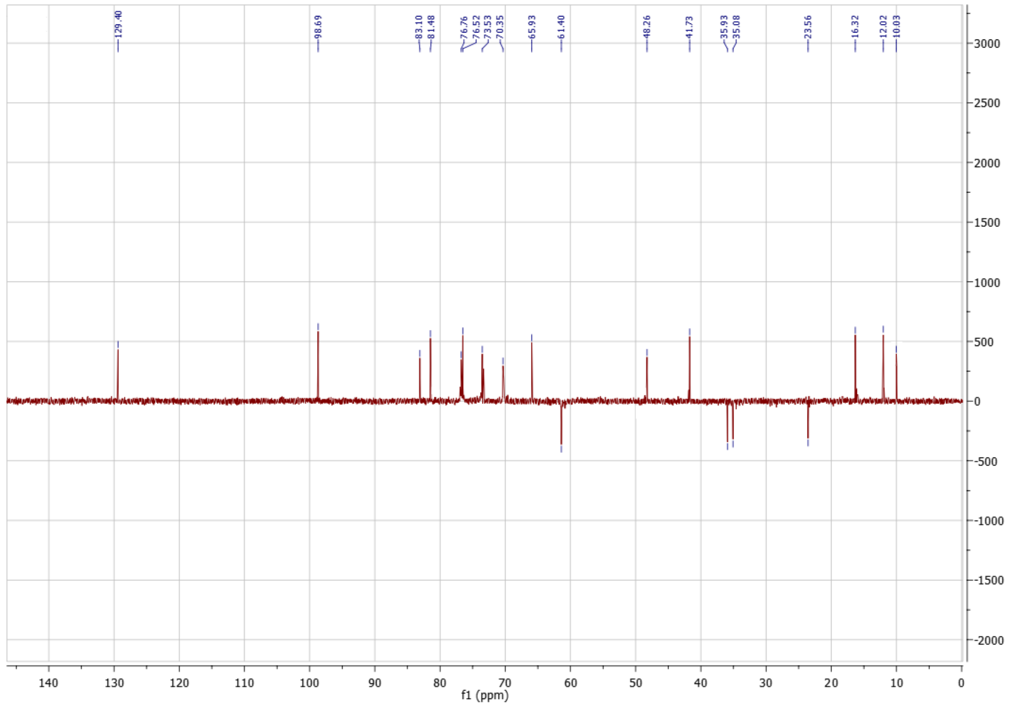

Figure S11. DEPT-135 NMR spectrum of Compound **2** in CD_3OD.

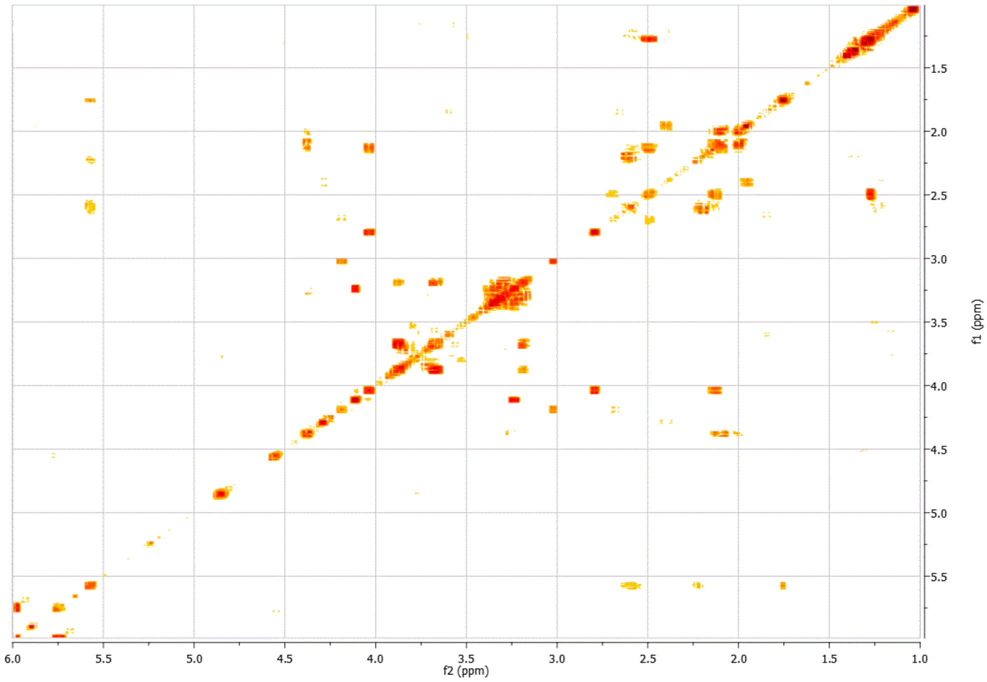

Figure S12. 2D COSY NMR spectrum of Compound **2** in CD_3OD.

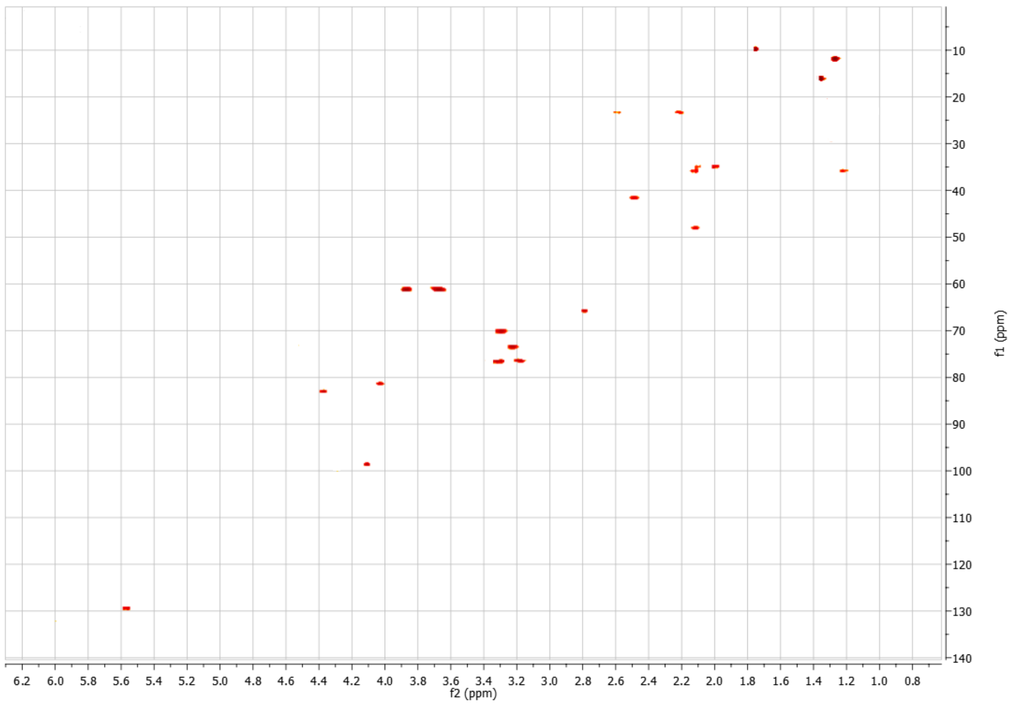

Figure S13. HSQC spectrum of Compound **2** in CD_3OD.

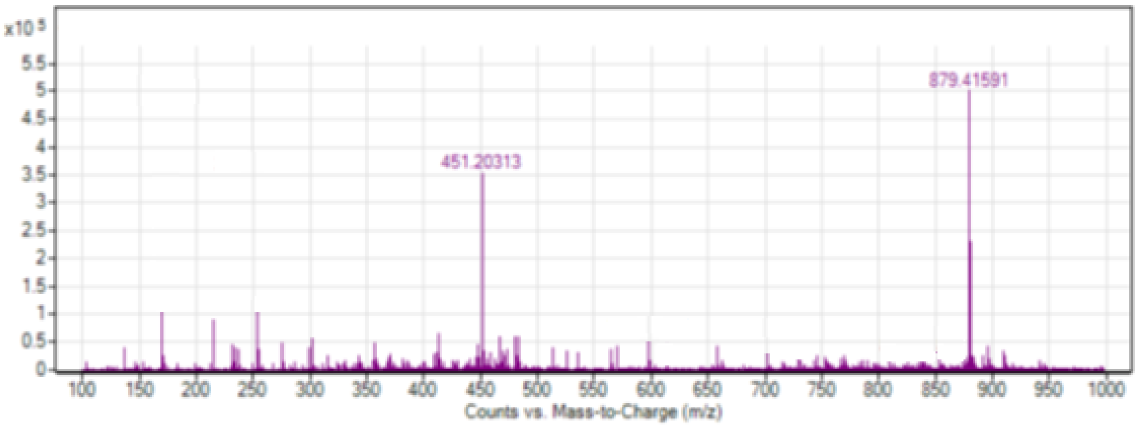

Figure S14. Positive ions ESI Mass spectrum of Compound **2**.

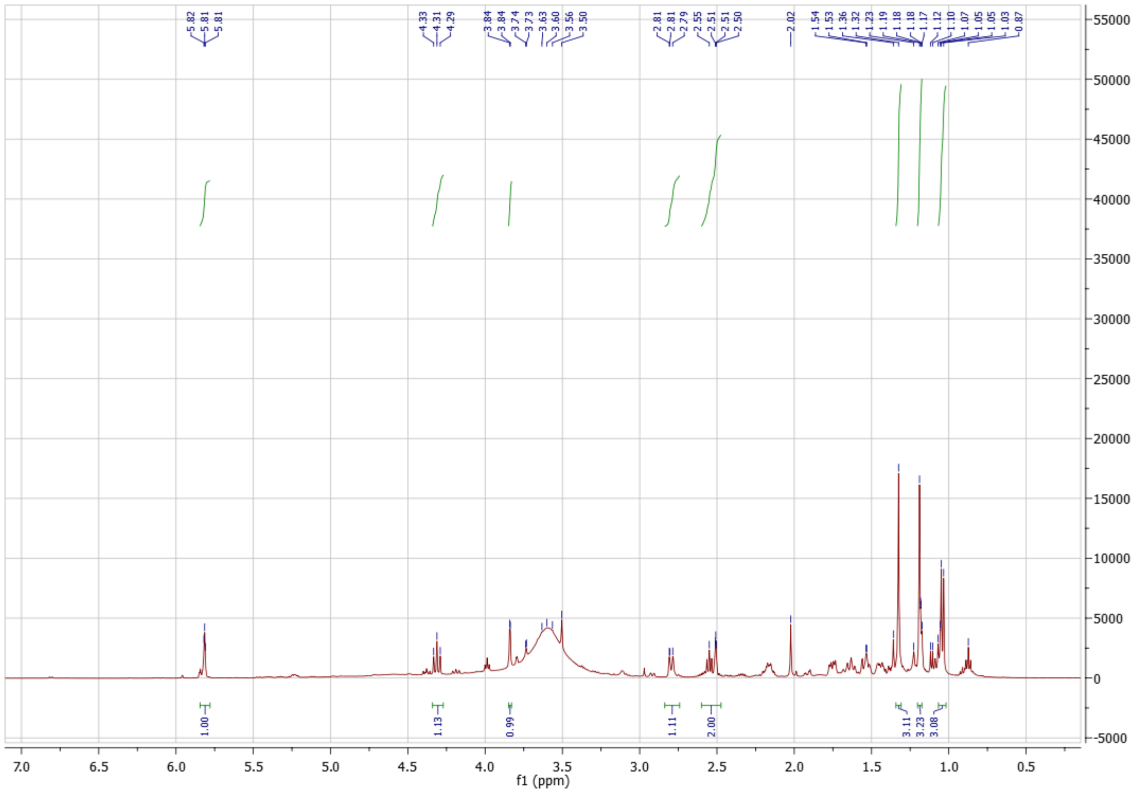

Figure S15. ^{1}H NMR spectrum of compound **3**.

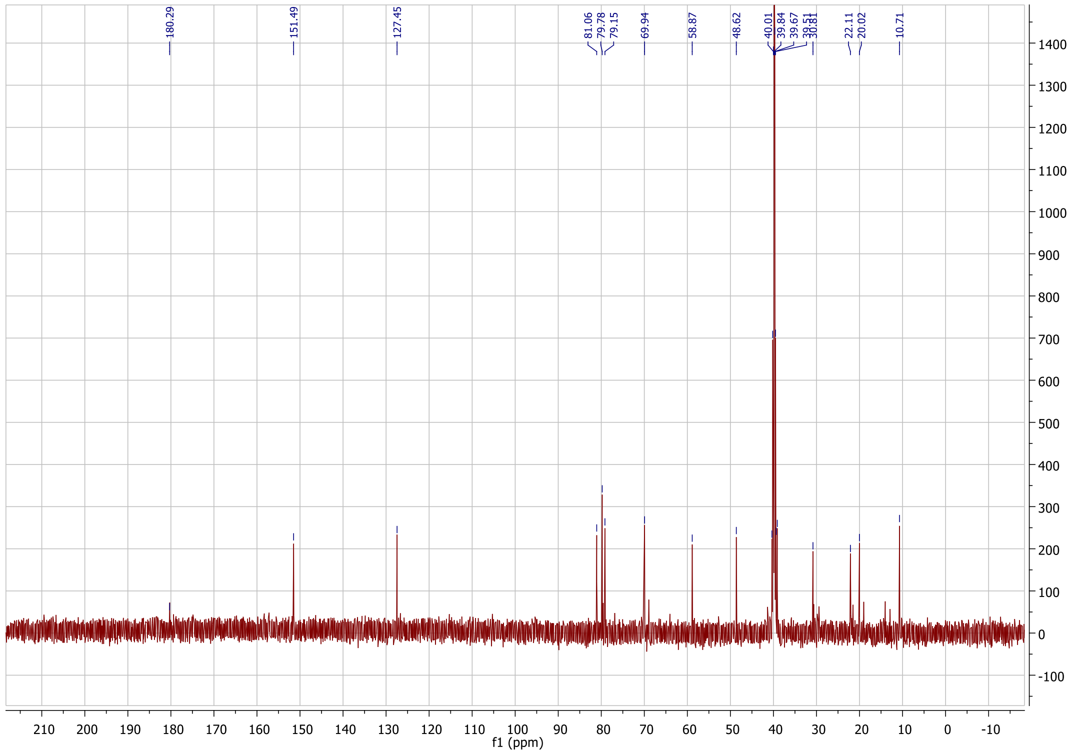

Figure S16. ^{13}C NMR spectrum of compound **3**.

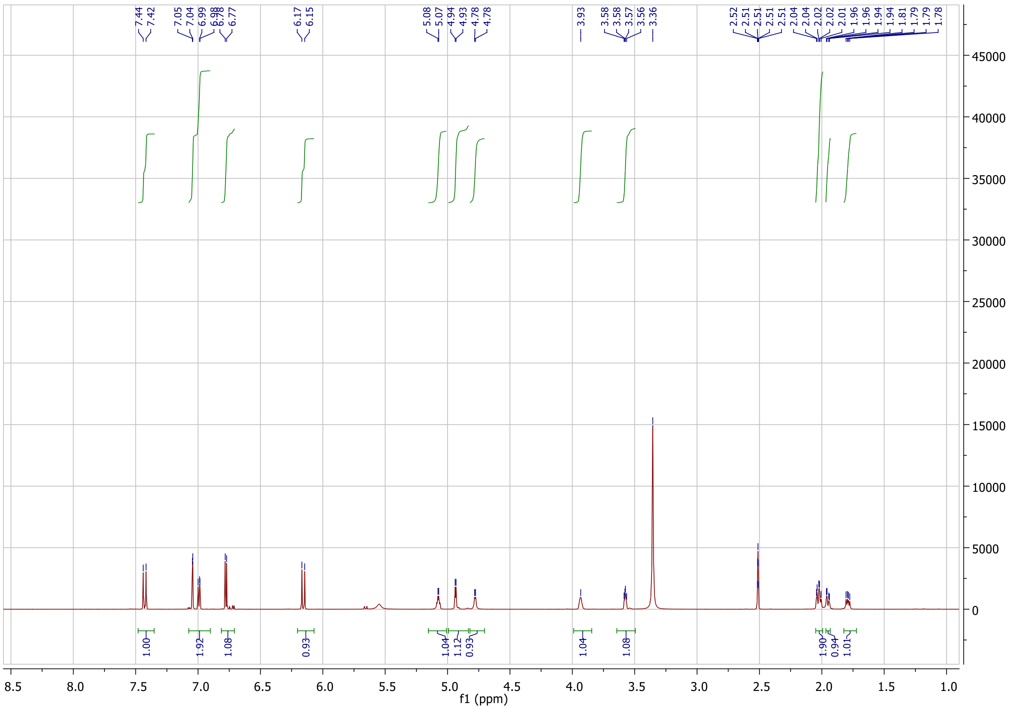

Figure S17. ^{1}H NMR spectrum of compound **4**.

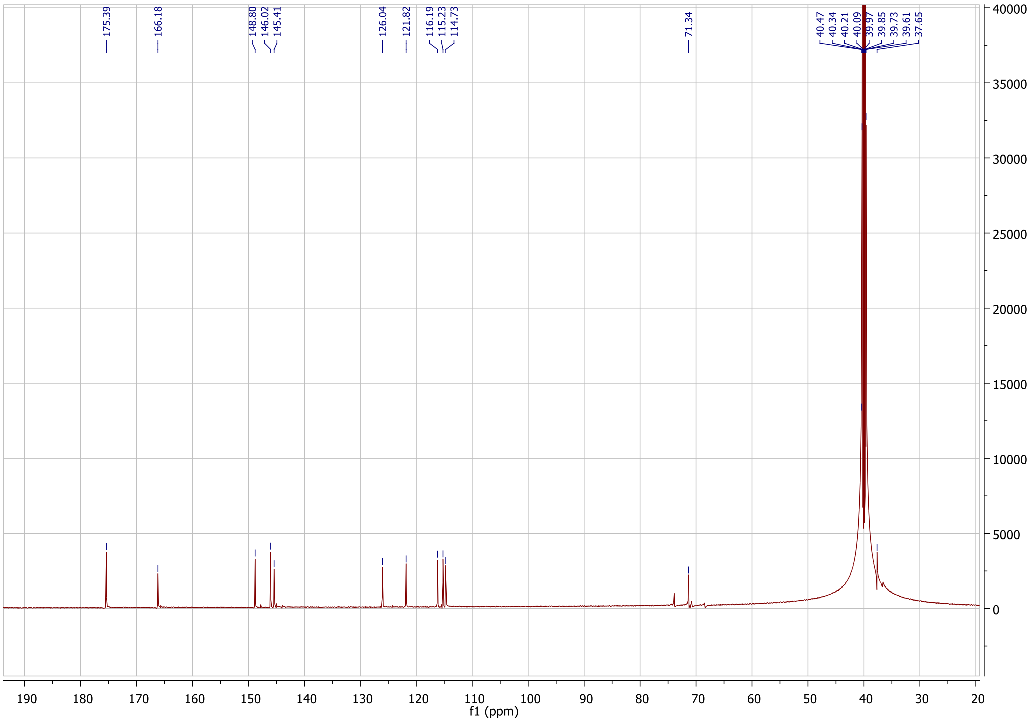

Figure S18. ^{13}C NMR spectrum of compound **4**.

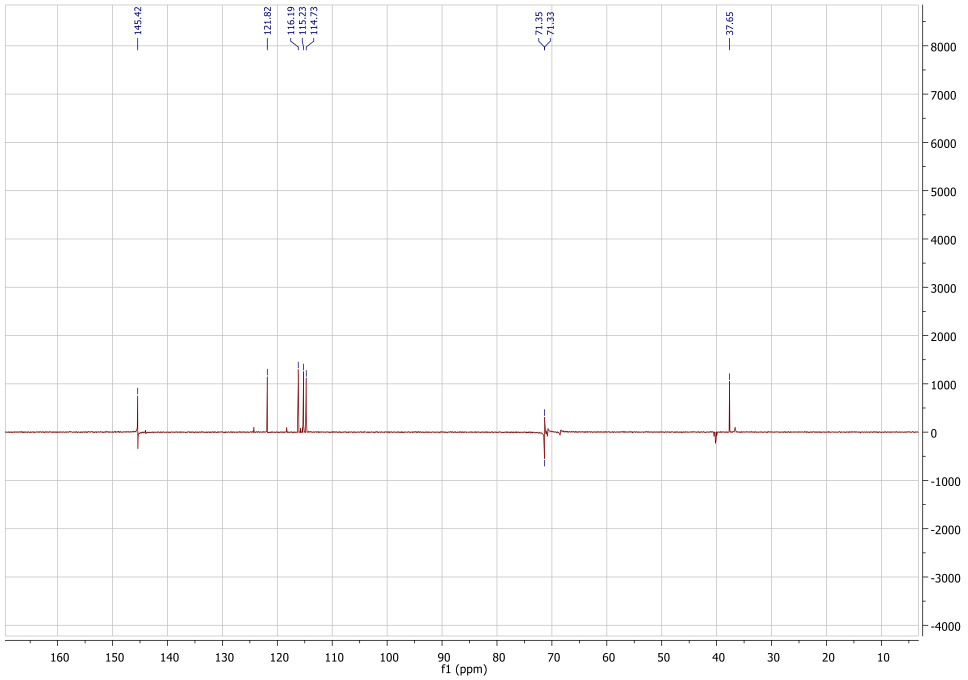

Figure S19. DEPT NMR spectrum of compound **4**.

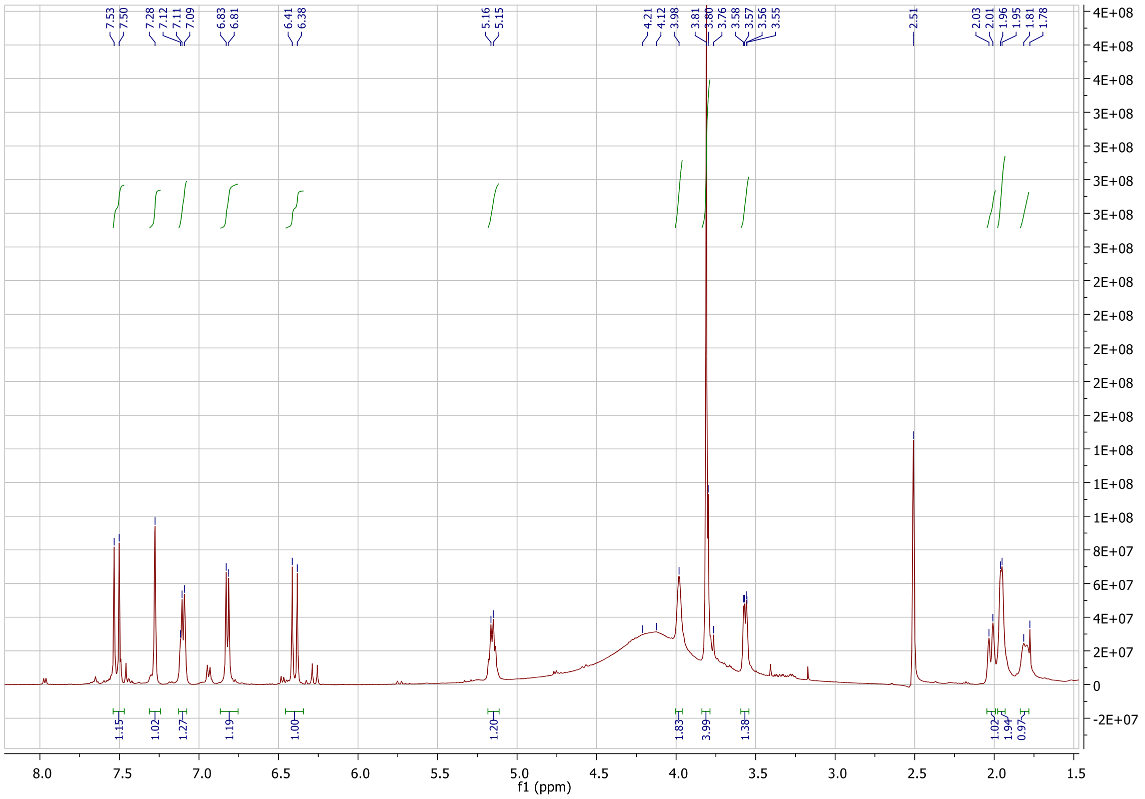

Figure S20. ^{1}H NMR spectrum of compound **5**.

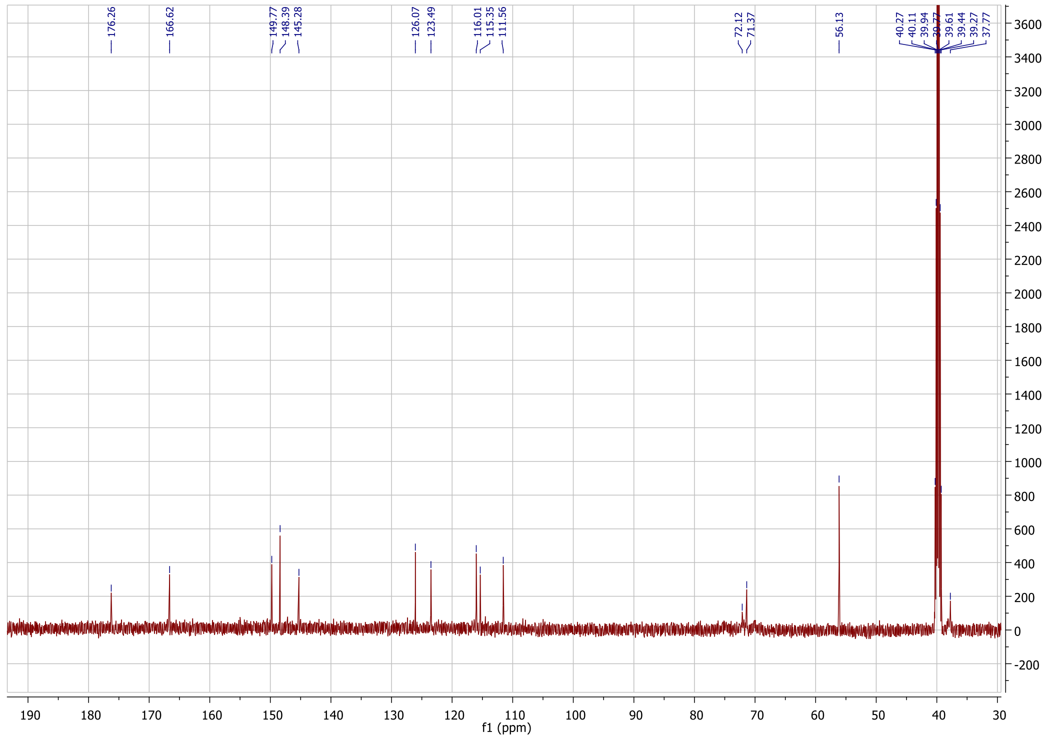

Figure S21. ^{13}C NMR spectrum of compound **5**.

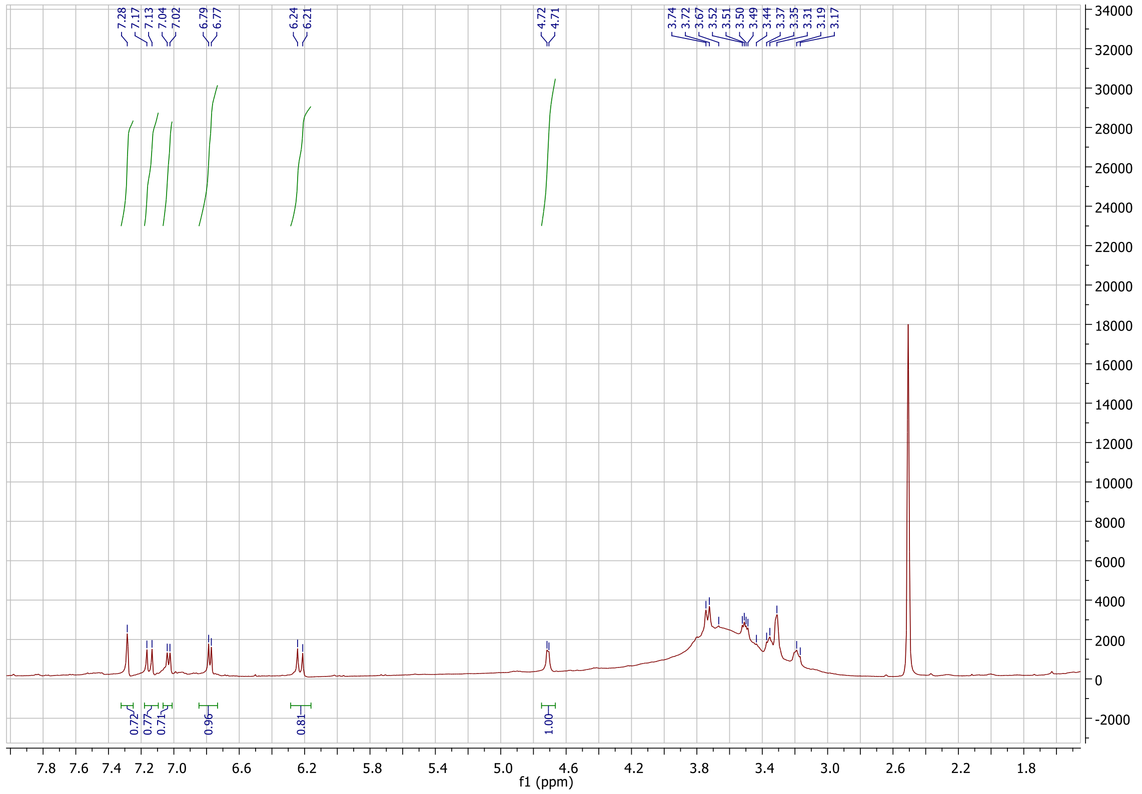

Figure S22. ^{1}H NMR spectrum of compound **6**.

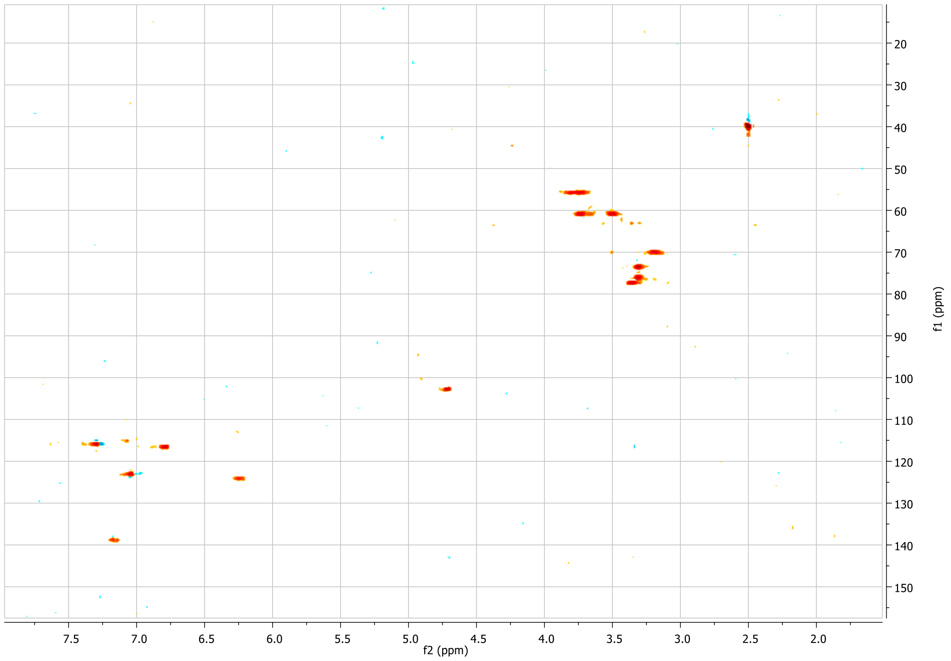

Figure S23. 2D HSQC NMR spectrum of compound **6**.
